# Community-based care provided by home-visiting nurses for families of individuals with mental illness, aimed at promoting family recovery in Japan

**DOI:** 10.1192/j.eurpsy.2025.1639

**Published:** 2025-08-26

**Authors:** M. Tanoue, M. Suzuki, S. Teraoka, S. Okubo, A. Watanabe

**Affiliations:** 1 Graduate School of Nursng, Chiba University, Chiba; 2 Faculty of Medical Technology, Teikyo University, Tokyo; 3 Institute of Ars Vivendi, Ritsumeikan University, Kyoto; 4 BMS Yokohama Inc., Kanagawa; 5 School of Nursing, Tokyo Health Care University, Chiba, Japan

## Abstract

**Introduction:**

Families of patients with mental illness in Japan face the stigma and the significant burden of caregiving. The average hospital stay for psychiatric patients in Japan was 276.3 days in 2022. Strengthening community support for patients with mental illness and their families requires targeted support that promotes recovery for both patients and their families.

**Objectives:**

To clarify the attitudes and perceptions of psychiatric home-visiting nurses toward family support in the community, aiming to empower families of patients with mental illness and identify key support issues for family recovery.

**Methods:**

1. A 30-item, web-based, anonymous questionnaire survey was conducted involving psychiatric home-visiting nurses who care for patients with mental illness The survey measured respondents’ background information and perceptions regarding the frequency and importance of family care practices.
2. Simple tabulations of the questionnaire items were performed, and the frequency of implementation was examined and related. 3) Items perceived as important but infrequently implemented were identified.

**Results:**

Sixty-six home-visiting nurses participated in the survey. The findings showed that 97% of respondents expressed interest in family support, 74% had family support experience, and 52% had attended family support training programs.

A significant correlation was observed between the perceived importance of family support and its perceived frequency of implementation across all items. The four items identified as important but less frequently implemented were:

Q08. Referring to other professional organizations for unresolved issues (22.7%).

Q11. Encouraging active participation from each patient and family member (20.6%).

Q12. Informing family members about the patient’s situation, so that they can work together to ensure the patient’s well-being (40.6%).

Q13. Encouraging patients to express appreciation to the family when needed (36.7%).

**Conclusions:**

The survey results indicate that family care items requiring patient engagement or collaborative decision-making, such as referrals to other agencies, were implemented less frequently. Home-visiting nurses face challenges in enhancing their skills and knowledge in areas such as family engagement, interagency collaboration, and discerning the appropriate scope and timing of interventions.

Strengthening these competencies will support more effective connections between patients with mental illness, their families, and community resources.

**Disclosure of Interest:**

None Declared

